# Geometry Preserving Sampling Method Based on Spectral Decomposition for Large-Scale Environments

**DOI:** 10.3389/frobt.2020.572054

**Published:** 2020-09-29

**Authors:** Mathieu Labussière, Johann Laconte, François Pomerleau

**Affiliations:** ^1^Université Clermont Auvergne, CNRS, SIGMA Clermont, Institut Pascal, Clermont-Ferrand, France; ^2^Northern Robotics Laboratory, Université Laval, Québec City, QC, Canada

**Keywords:** sampling, spectral decomposition, large-scale environments, tensor voting, iterative closest point (ICP), registration, 3D mapping, lidar

## Abstract

In the context of 3D mapping, larger and larger point clouds are acquired with lidar sensors. Although pleasing to the eye, dense maps are not necessarily tailored for practical applications. For instance, in a surface inspection scenario, keeping geometric information such as the edges of objects is essential to detect cracks, whereas very dense areas of very little information such as the ground could hinder the main goal of the application. Several strategies exist to address this problem by reducing the number of points. However, they tend to underperform with non-uniform density, large sensor noise, spurious measurements, and large-scale point clouds, which is the case in mobile robotics. This paper presents a novel sampling algorithm based on spectral decomposition analysis to derive local density measures for each geometric primitive. The proposed method, called Spectral Decomposition Filter (SpDF), identifies and preserves geometric information along the topology of point clouds and is able to scale to large environments with a non-uniform density. Finally, qualitative and quantitative experiments verify the feasibility of our method and present a large-scale evaluation of SpDF with other seven point cloud sampling algorithms, in the context of the 3D registration problem using the Iterative Closest Point (ICP) algorithm on real-world datasets. Results show that a compression ratio up to 97 % can be achieved when accepting a registration error within the range accuracy of the sensor, here for large scale environments of less than 2 cm.

## 1. Introduction

**Figure 1:**
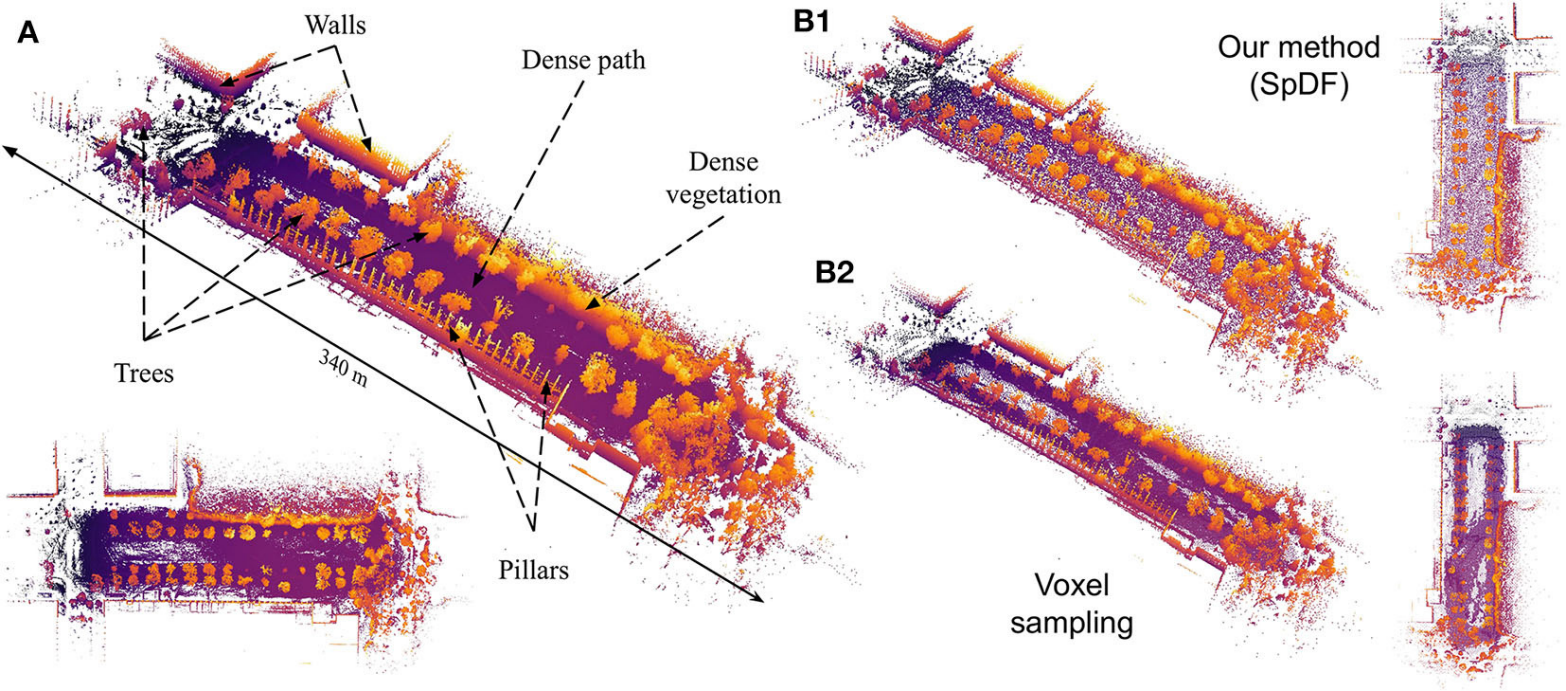
**(A)** This large-scale map, containing more than 4.65 million points, presents structured (walls, pillars) and unstructured (trees, vegetation) elements with varying densities. Color represents the elevation, the brighter the color, the higher the *z*-coordinate. Views of the reduced map to 100 k points (i.e., a compression ratio of 97.85 %) by spatial sampling **(B2)** with a spatial extent of 0.8 m, keeping points regularly given the spatial decomposition of the point but losing some geometric details; by our proposed method **(B1)** where the density is uniform and more geometric details have been preserved.

Light Detection And Ranging (Lidar) sensors has recently been widely democratized in robotics applications. Indeed, these sensors are able to acquire an efficient representation of the environment (i.e., a point cloud), which can be used in localization algorithms, 3D mapping or environments inspection (Stumm et al., 2012). Prior work on lidar-based registration algorithms have been recently used to create larger and larger 3D maps (Pomerleau et al., 2014). As an example, Figure 1 shows the map of the *Grand Axe* of Laval University campus, where only a few minutes of data collection lead to a number of points at the limit of the real-time computation capability. The sensor used for this map, the Velodyne HDL-32E, yields up to 1.39 million points per second. Although pleasing to the eye, dense maps are not necessarily tailored for practical applications. Indeed, such point clouds are heavy to process and transmit: in scenarios where the processing power and bandwidth are critical resources, point clouds need to be compressed or sampled before any other manipulation. For instance, search & rescue missions often lead the robot in areas where the bandwidth is very narrow. As the tele-operator needs quick feedbacks on where the robot is heading, having access to point clouds with low memory footprint to send through the network without losing information is a necessity.

For any of these tasks, the robot needs to merge the different acquired point clouds to create a dense 3D map as it is a necessary information for most of robotics applications. The Iterative Closest Point (ICP) is one of the main algorithms for creating such maps, introduced by Besl and McKay (1992) and Chen and Medioni (1992). It is still considered a strong solution for registration in mobile robotics as shown by Pomerleau et al. (2015). As we want to sample the point cloud, we need to make sure that such action does not hinder the ICP process, since the contrary would lead to noisy, impractical maps. On the one hand, limiting the growth by reducing the number of points will enlarge the spectrum of real-time applications. On the other hand, reducing the number of points too aggressively can lead to unworkable localization and mapping and might discard potential critical information. For example, keeping the edges of objects while doing surface inspection is essential to detect cracks, whereas sampling very dense areas of very little information such as the ground would lead to a substantial compression. Under these considerations, geometric primitives seem a good approach to capture the details along the topology (Stumm et al., 2012). This information can be retrieved by the methodology of Tensor Voting introduced by Guy and Medioni (1997) and Medioni et al. (2000).

As opposed to solutions for registration-based object reconstruction, we will consider large-scale 3D environments, which are still challenging even for the state-of-the-art sampling methods, because of the uneven density coming from the radial distribution of lidars. Given these working hypotheses, contributions of this paper are 2-fold:

1. A novel sampling method, called Spectral Decomposition Filter (SpDF), based on spectral decomposition analysis. This method identifies and preserves geometric information along the topology of point clouds and is scalable to large environments.
2. A large-scale evaluation of current sampling strategies relying on more than 2.45 million registrations in different types of environments (indoor/structured, outdoor/unstructured), including large-scale outdoors environments from real-world datasets.

**Figure 2:**
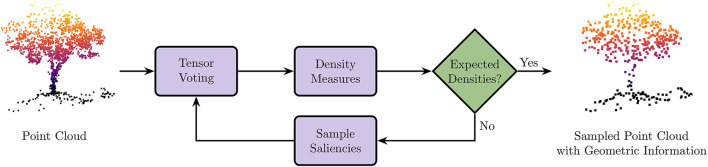
Overview of our sampling strategy. Point cloud is given as input. First, we identify the geometric primitives along with their saliencies using the Tensor Voting framework. Then, we derive density measures from saliencies: if the density for each geometric primitive is less than the desired density, we stop; else we sub-sample each geometric primitive, and re-iterate. As output, we have a uniform sampled point cloud enhanced with geometric information.

A visual overview of our sampling strategy is given by the Figure 2. A point cloud is given as input. First, we identify the geometric primitives along with their saliencies using the tensor voting framework. Then, we derive density measures from saliencies: if the density for each geometric primitive is less than the desired density, we stop; else we sub-sample each over-represented geometric primitive, and re-iterate. As output, we have a uniform sampled point cloud enhanced with geometric information.

This paper is organized as follows. First, we review the existing sampling strategies of the literature in section 2. Secondly, we briefly summarize the theory of tensor voting for completeness in section 3. Then, we show how to derive new density measures from the output of the tensor voting process in section 4, and how these new measures are used to efficiently sample point clouds in section 5. Finally, we present our experimental setup in section 6 and analyze the results of our method along with the evaluation of seven other sampling strategies from the state-of-the-art in section 7.

## 2. Related Works

A point-sampled surface is a good representation for analyzing the properties of 3D shapes (Alexa et al., 2001). Unfortunately, most point clouds obtained in robotics context are noisy, sparse, large, and have an uneven density. An important step during the process of analyzing point clouds is to remove noise and outliers. This can be done using filtering algorithms. An extensive review of these algorithms has been realized by Han et al. (2017). Point cloud simplification is related to the problem addressed by the computer vision field but aims to accelerate graphic rendering. A lot of methods based on meshing are used to address this problem (Hoppe, 1996; Li and Zhu, 2008). Technically, these methods can be directly extended to point cloud representation, but most of the algorithms perform an expensive dataset meshing pre-step. A review and comparison of mesh simplification algorithms has been done by Cignoni et al. (1998). Mesh-free algorithms have also been developed to directly simplify point clouds. For instance, Pauly et al. (2002) introduced, analyzed and quantitatively compared a number of surface simplification methods for point-sampled geometry. More recently, Leal et al. (2017) presented a novel method for point cloud simplification using an estimated local density of the point cloud, requiring no additional mesh estimation procedure. Most of these algorithms have been designed in context of computer graphics applications. They compare themselves only with few others strategies, and rely most of the time on evaluations conducted on small, uniform, dense, and complete point clouds such as object models. These working hypotheses usually break in the case of most robotics applications, where point clouds are noisy, incomplete, and sparse. In addition, the sampling process can be addressed by signal processing strategies. Indeed, a point cloud can be considered as a manifold sample. Pauly and Gross (2001) introduced the concept of local frequencies on geometry in order to be able to use all existing signal processing algorithms. Oztireli et al. (2010) proposed a new method to find optimal sampling conditions based on spectral analysis of manifolds. However, these methods stand under the hypothesis of smooth manifolds, which is usually not the case in maps acquired with lidar sensors in robotics, and rely on evaluations conducted on object models data only.

Sampling algorithms aim to decrease the complexity of point cloud processing (e.g., the computation time) by reducing the number of input points. For instance, ICP algorithm complexity depends on the number of point to process. There are different strategies for points selection that can be categorized as global methods (e.g., uniform and random sampling, spatial sampling), local methods (e.g., using geometric information or density information), and feature-based methods. Feature-based methods such as Fast Point Feature Histogram (FPFH) introduced by Rusu et al. (2009), or Feature-Preserved Point cloud Simplification (FPPS) presented by Zhang et al. (2019) use features which describe the local geometry around a point. It reduces then the number of points by grouping them to describe the neighborhood. These methods provide improvements only with point clouds where features are distinctive which is hard to obtain with noise or incomplete data (Mellado et al., 2014). Hence, this paper will only focus on global and local methods. The most wildly used methods are based on octree or voxel representations of point clouds (Schnabel and Klein, 2006; Elseberg et al., 2013; Hornung et al., 2013; Fossel et al., 2017). They take into consideration the spatial distribution of the points by regularly partitioning the space into cells aligned with the *xyz*-basis. Then, they reduce the number of points by taking the most representative point in each cell, e.g., the centroid. Spatial segmentation methods however do not take into account distinctiveness between the points within cells. For instance, when several geometric features are present in the same cell, the information is lost, which is the case of details in dense areas. They lack of fine grained control over the number of output points and over the level of details.

Another category of methods analyzes geometric primitives to sample relevant points in point clouds. Adaptive sampling strategy based on local density estimation has been proposed by Al-Durgham (2014) in context of registration. Al-Rawabdeh et al. (2020) further extended the latter by sub-sampling on the Gaussian sphere, where normals have been estimated by Principal Component Analysis (PCA), but evaluated their method only on precise point clouds and only against random sampling. Rusinkiewicz and Levoy (2001) proposed a method based on normals analysis named Normal-Space Sampling (NSS). Points are sampled uniformly according to their normal orientations. They state that it helps convergence for scenes with small, sparse features but by their nature, normals cannot help to handle rotational uncertainties. Rodolà et al. (2015) defined the concept of relevance based on curvature to sample points but such primitives are often noisy and must be processed carefully (Kalogerakis et al., 2009). They compared themselves with the latter strategy and against uniform sampling, in context of object model registration through ICP. Kwok (2018) extended the work on normal space to handle rotational error by introducing a dual normal space to constrain both translation and rotation. He evaluated his method Dual Normal-Space Sampling (DNSS) against several methods, including feature-based and normal-based method, but only on uniformly sampled mesh models. Both NSS and DNSS do not take into account the spatial distribution of the sampling points as they only analyze the normals distributions. Points are not guaranteed to be kept uniformly in space, leading to less accurate results in large-scale sparse point clouds. Gelfand et al. (2003) presented a method based on covariance analysis, Covariance Sampling (CovS), to perform stability analysis in order to select geometrically stable points that can bind the rotational component as well as the translation. An improvement of CovS has been proposed in the context of manufacturing by Kwok and Tang (2015). No evaluation have been conducted on real-world large-scale point cloud for these methods. The authors stated that their proposed approach may suffer from high levels of noise, as large noises make some originally smooth areas strongly constraining. Given our results, previous methods cannot handle noisy, large-scale and density-varying point clouds when they are used to reduce the number of points. Eventually, similar to our method but subsequent to our previous work (Labussière et al., 2018), Ervan and Temeltas (2019) also proposed a sampling algorithm based on a modified tensor voting framework to preserve geometric primitives while down sampling dense areas taking most salient points as representatives. Only qualitative results are given on only one scan, and no comparison have been made. The method presented in this paper is able to both reduce the number of point and retrieve the geometric information in large sparse noisy point clouds. Contrarily to other strategies, we presents a large-scale evaluation of these strategies along with our method in context of large-scale 3D environments.

Although several new strategies have been proposed in the recent years, the most used point cloud processing software such as CloudCompare, the Point Cloud Library (PCL), or libpointmatcher, a popular modular library implementing the Iterative Closest Point (ICP), mostly still rely on either random, uniform or spatial sampling to reduce the number of points.

## 3. Tensor Voting: Theory

Medioni et al. (2000) introduced Tensor Voting (TV) as a methodology to infer geometric information (e.g., surface, curve, and junction descriptions) from sparse 3D data^1^. The algorithm is based on tensor calculus for data representation and tensor voting for data communication. Theory related to TV will be summarized in this section for completeness.

### 3.1. Tensor Representation

To capture the first order differential geometry information and its saliency, each datum can be represented as a second order symmetric tensor in the normal space. In 3D, such a tensor can be visualized as an ellipsoid with a shape that defines the nature of the information and a scale that defines the saliency of this information. A second order symmetric tensor ***K*** is fully described by its associated spectral decomposition using three eigenvectors ***e***_1_, ***e***_2_, and ***e***_3_, and three corresponding ordered positive eigenvalues λ_1_ ≥ λ_2_ ≥ λ_3_. This tensor can be decomposed in three basis tensors, resulting in

(1) $$\begin{array}{l} K=\left(\lambda_{1}-\lambda_{2}\right)S+\left(\lambda_{2}-\lambda_{3}\right)P+\lambda_{3}B, \end{array}$$

with

(2) $$\begin{array}{l} \;S=e_{1}e_{1}^{T},\\ \;P=\overset{2}{\underset{d=1}{\sum }}e_{d}e_{d}^{T},\\ B=\overset{3}{\underset{d=1}{\sum }}e_{d}e_{d}^{T}, \end{array}$$

where ***S*** describes the stick tensor, ***P*** the plate tensor, and ***B*** the ball tensor.

### 3.2. Voting Process

The main goal of Tensor Voting is to infer information represented by the tensor ***K***_*i*_ at each position ***x***_*i*_ by accumulating cast vote **V** from its neighborhood N, following

(3) $$\begin{array}{l} K_{i}=\underset{x_{j}\in N\left(x_{i}\right)}{\sum }\text{V}\left(x_{i},x_{j}\right). \end{array}$$

This process can be interpreted as a convolution with a predefined aligned voting field. The voting fields encode the basis tensors and are derived from the 2D stick field by integration (see Medioni et al., 2000 for more details). Each input point is encoded into a tensor. First, if no direction is given, the tensor encodes a unit ball ***B***. Second, if tangents are provided, the tensor encodes a plate ***P***. Finally, if normals are available, the tensor encodes a stick ***S***. In a case where no direction is given, a first pass of refinement is done to derive the preferred orientation information. Each tensor then broadcasts each of its independent elements using an appropriate tensor field:

(4) $$\begin{array}{l} \text{V}\left(x_{i},x_{j}\right)={\text{V}}_{S}\left(x_{i},x_{j}\right)+{\text{V}}_{P}\left(x_{i},x_{j}\right)+{\text{V}}_{B}\left(x_{i},x_{j}\right), \end{array}$$

where **V**_***S***_ (resp. **V**_***P***_ and **V**_***B***_) is the vote generated by the tensor field associated to ***S*** (resp. ***P*** and ***B***).

### 3.3. Vote Interpretation

The resulting generic second order symmetric tensor ***K*** is then decomposed into elementary components to extract the saliencies and the preferred direction. The interpretation of these values is given in Table 1. We can then infer geometric primitives, but the procedure to extract the salient features corresponding to local maxima of the three saliency maps will not be discussed here.

**Table 1:** Interpretation of saliencies and preferred directions obtained by the tensor voting framework, where the λ_*d*_ are the eigenvalues associated to the eigenvectors ***e***_*d*_ obtained from spectral decomposition of the resulting tensors. Predominating saliency determines the affected geometric primitive.

	**Geom. Primitive**	**Tensor**	**Saliency**	**Normals**
Surface-ness	Surface	Stick ***S***	λ_1_ − λ_2_	***e***_1_
Curve-ness	Curve	Plate ***P***	λ_2_ − λ_3_	***e***_1_, ***e***_2_
Point-ness	Junction	Ball ***B***	λ_3_	***e***_1_, ***e***_2_, ***e***_3_

### 3.4. k-Nearest Neighbors Closed Form Tensor Voting

Although tensor voting is a robust technique for extracting perceptual information from point clouds, the complexity of its original formulation makes it difficult to use in robotics applications. We use the closed-form (CFTV) formulation proposed by Wu et al. (2012) for efficiency. The generic second order symmetric tensor is then computed given

(5) $$\begin{array}{l} K_{i}=\underset{x_{j}\in N\left(x_{i}\right)}{\sum }{\text{S}}_{ij}\text{ with }{\text{S}}_{ij}=c_{ij}{\text{R}}_{ij}K_{j}{\text{R}}_{ij}^{\prime }, \end{array}$$

and

(6) $$\begin{array}{ll} {\text{R}}_{ij}=\left(\text{I}-2r_{ij}r_{ij}^{T}\right), & {\text{R}}_{ij}^{\prime }=\left(\text{I}-\frac{1}{2}r_{ij}r_{ij}^{T}\right){\text{R}}_{ij}^{T},\\ r_{ij}=\frac{x_{i}-x_{j}}{∥x_{i}-x_{j}∥}, & c_{ij}=\exp\left(-\frac{∥x_{i}-x_{j}{∥}^{2}}{\sigma }\right), \end{array}$$

where *c*_*ij*_ is a decay function and controls the strength of the vote given the distance between the two positions and the scale parameter σ; ***r***_*ij*_ is the normalized vector from ***x***_*j*_ in the direction of ***x***_*i*_; and N is the neighborhood retrieved using an efficient *k*-Nearest Neighbors (*k*-NN) search (e.g., with a *kD-tree*). As the input is generally not oriented, we still have to do a first pass by encoding ***K***_*j*_ as a unit ball to derive a preferred direction. Then, we do a second pass by encoding points with the tensors previously obtained, but with the ball component disabled as suggested by Wu et al. (2012), such as ***K***_*j*_ = (λ_1_ − λ_2_)***S***_*j*_ + (λ_2_ − λ_3_)***P***_*j*_. Once the generic tensor is computed, we decompose and interpret it as shown above.

## 4. Derivation of Density Measures

Based on tensor voting theory, this paper presents a novel density measure for each geometric primitive. By doing a first pass of TV using the closed-form with an *k*-NN search (5), we are able to derive more information from the tensors. In fact we can show that 0 ≤ λ_*d*_ ≤ *k*, ∀*d* ∈ {1, 2, 3}, where *k* is the number of neighbors. As the strength of the vote through the decay function is directly dependent on the distance, we have λ_*d*_ = *k* when all neighbors are at a distance δ = 0. Given this observation, the lambdas can be considered as an indicator of local density.

In the following, the λ_*d*_ are normalized by *k*. We can compute the expected normalized vote strengths ξ_*D*_ at a position where the density would be uniform in a *D*-hyperball of radius ρ to derive the density measures. The strength of the vote *c*_*ij*_ is only dependent on the distance δ between ***x***_*i*_ and ***x***_*j*_ (6) such as $\delta =∥x_{i}-x_{j}{∥}^{2}$. Therefore only the decay function c(δ) = exp (−δ^2^/σ) is taken into account. We compute the expectation of the decay function given points following a uniform spatial distribution in a *D*-hyperball of radius ρ. In order to achieve this distribution, we can generate samples of the distance δ through the random variables $X\sim U_{\left[0,1\right]}$, where $U_{\left[0,1\right]}$ is the uniform distribution between 0 and 1, by using the inverse of the cumulative distribution function (CDF) corresponding to the surface area of this hyperball. A random sample is generated by mapping random numbers in the range [0, 1] through the application $\delta \left(\text{x}\right)=\rho \cdot x^{\frac{1}{D}}$. We then compute the expected value of this distribution such as

(7) $$\begin{array}{l} \text{E}\left[\text{c}\left(\delta \left(X\right)\right)\right]=\int_{-\infty }^{\infty }{\text{pdf}}_{X}\left(x\right)\cdot \text{c}\left(\delta \left(x\right)\right)\text{d}x\\ \;=\frac{D}{2}{\left(\frac{\rho^{2}}{\sigma }\right)}^{-\frac{D}{2}}\left(\Gamma \left(\frac{D}{2}\right)-\Gamma \left(\frac{D}{2},\frac{\rho^{2}}{\sigma }\right)\right), \end{array}$$

where σ is the scale of the vote, Γ(·) is the gamma function and Γ(·, ·) is the incomplete gamma function. For *D* ∈ {1, 2, 3}, the expected kernel strengths are given by

(8) $$\begin{array}{l} \xi_{1}=\frac{1}{4\rho }\;\sqrt{\pi \sigma }\text{ erf}\left(\frac{\rho }{\sqrt{\sigma }}\right)\\ \xi_{2}=\frac{\sigma }{\rho^{2}}\;\left(1-\exp\left(-\frac{\rho^{2}}{\sigma }\right)\right)\\ \xi_{3}=\frac{3\sigma }{4\rho^{3}}\;\left(\sqrt{\pi \sigma }\text{ erf}\left(\frac{\rho }{\sqrt{\sigma }}\right)-2\rho \exp\left(-\frac{\rho^{2}}{\sigma }\right)\right), \end{array}$$

where erf(·) is the Gauss error function, ξ_1_ is the expected strength in the plate case (*D* = 1), ξ_2_ is the expected strength in the stick case (*D* = 2) and ξ_3_ is the expected strength in the ball case (*D* = 3).

**Figure 3:**
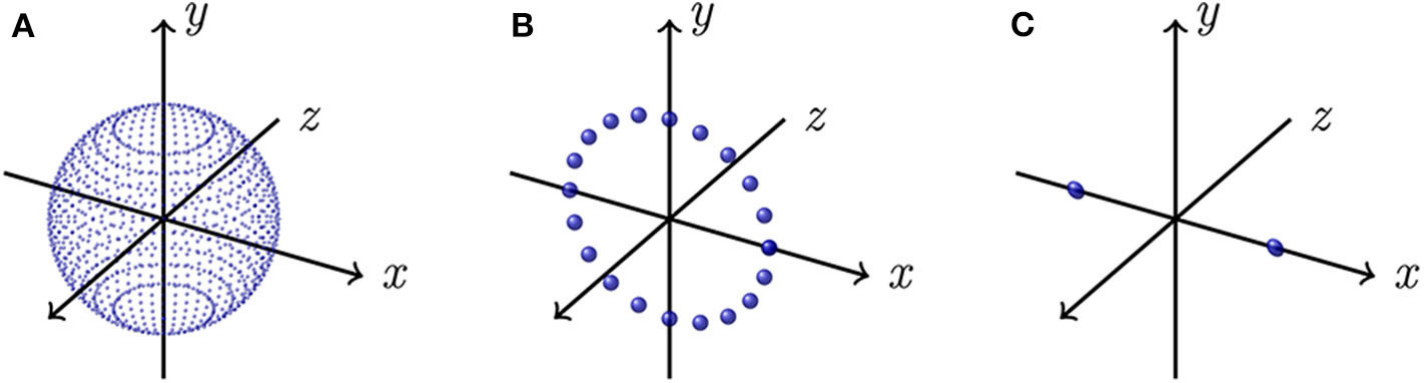
Ideal simplified voting situations. **(A)** All points are uniformly distributed on a sphere (*D* = 3); **(B)** All points are uniformly distributed on a circle lying in the *xy*-plane (*D* = 2); **(C)** All points are uniformly distributed on the endpoints of a line segment along the *x*-axis (*D* = 1).

The associated eigenvalues ${\hat{\lambda }}_{d}$ can be derived if we consider, for each component, the ideal cases illustrated by Figure 3 where each voter strength is ξ_*D*_. By developing (5) with *c*_*ij*_ = ξ_*D*_, considering an infinite number of neighbors, thus taking ***r***_*ij*_ as the integral variable on the considered domain (i.e., all possible orientations in *D* dimensions), we compute for each case the expected eigenvalues ${\hat{\lambda }}_{d}$. For instance, in the case where points are uniformly distributed on a sphere (i.e., *D* = 3 as illustrated by Figure 3A) of radius such that *c*_*ij*_ = ξ_3_, the integral variable is given by the normalized vector $r\;=\;{\left[\begin{array}{ccc} \sqrt{1-u^{2}}\cos\left(\theta \right) & \sqrt{1-u^{2}}\sin\left(\theta \right) & u \end{array}\right]}^{T}$ with (θ, *u*) ∈ [0, 2π] × [−1, 1], and the resulting normalized tensor by

(9) $$\begin{array}{l} \bar{K}=\eta \int_{\Gamma }\xi_{D}\left(\text{I}-2rr^{T}\right)\left(\text{I}-\frac{1}{2}rr^{T}\right){\left(\text{I}-2rr^{T}\right)}^{T}\text{d}r\\ \;=\frac{1}{4\pi }\int_{0}^{2\pi }\int_{-1}^{1}\xi_{3}\left(\text{I}-2rr^{T}\right)\left(\text{I}-\frac{1}{2}rr^{T}\right){\left(\text{I}-2rr^{T}\right)}^{T}\text{d}u\text{ d}\theta \\ \;=\frac{\xi_{3}}{4\pi }\left[\begin{array}{ccc} \frac{20}{6}\pi & 0 & 0\\ 0 & \frac{20}{6}\pi & 0\\ 0 & 0 & \frac{20}{6}\pi \end{array}\right]=\left[\begin{array}{ccc} \frac{5}{6}\xi_{3} & 0 & 0\\ 0 & \frac{5}{6}\xi_{3} & 0\\ 0 & 0 & \frac{5}{6}\xi_{3} \end{array}\right]=\left[\begin{array}{ccc} {\hat{\lambda }}_{3} & 0 & 0\\ 0 & {\hat{\lambda }}_{2} & 0\\ 0 & 0 & {\hat{\lambda }}_{1} \end{array}\right] \end{array}$$

where Γ is the integration domain of ***r***, and $\eta ={\left(\int_{\Gamma }\text{d}r\right)}^{-1}$ is the normalization constant since the eigenvalues are normalized by the number of neighbors. Finally, we have ${\hat{\lambda }}_{1}={\hat{\lambda }}_{2}={\hat{\lambda }}_{3}=\frac{5}{6}\;\xi_{3}$ and therefore the expected point-ness saliency is $\frac{5}{6}\;\xi_{3}$. In a similar fashion for the case *D* = 2 illustrated by Figure 3B, points are uniformly distributed on a circle of radius such that *c*_*ij*_ = ξ_2_, the integral variable is given by $r={\left[\begin{array}{ccc} \cos\left(\theta \right) & \text{sin}\left(\theta \right) & 0 \end{array}\right]}^{T}$ with θ ∈ [−π, π], and the expected surface-ness saliency is given by $\frac{1}{4}\;\xi_{2}$. For the case *D* = 1 illustrated by Figure 3C, points are uniformly distributed on line segment endpoints along the *x*-axis, of length such that *c*_*ij*_ = ξ_1_, we only have to consider the integral variable $r\in \left\{{\left[\begin{array}{ccc} 1 & 0 & 0 \end{array}\right]}^{T},\;{\left[\begin{array}{ccc} -1 & 0 & 0 \end{array}\right]}^{T}\right\}$, and the expected curve-ness saliency is given by $\frac{1}{2}\;\xi_{1}$.

We are now able to interpret the saliencies obtained by a first pass of the closed-form TV where every point has been encoded as ball tensor (i.e., ***K***_*j*_ = ***I***, ∀*j*) as a measure of local density. We can therefore compare the values with the expected saliencies, summarized in Table 2, to control the density of each geometric primitive.

**Table 2:** Expected eigenvalues and saliencies in the case of a uniform density in a *D*-hyperball, which can be interpreted as density measures and allow to control the density of each geometric primitive.

	**D**	**Eigenvalues**	**Saliency**
Curve-ness (***P***)	1	${\hat{\lambda }}_{1}={\hat{\lambda }}_{2}=\xi_{1}\text{and}{\hat{\lambda }}_{3}=\frac{1}{2}\;\xi_{1}$	$\frac{1}{2}\;\xi_{1}$
Surface-ness (***S***)	2	${\hat{\lambda }}_{1}=\xi_{2}\text{and}{\hat{\lambda }}_{2}={\hat{\lambda }}_{3}=\frac{3}{4}\;\xi_{2}$	$\frac{1}{4}\;\xi_{2}$
Point-ness (***B***)	3	${\hat{\lambda }}_{1}={\hat{\lambda }}_{2}={\hat{\lambda }}_{3}=\frac{5}{6}\;\xi_{3}$	$\frac{5}{6}\;\xi_{3}$

## 5. Spectral Decomposition Filter (SpDF): Overview

The method presented in this article, namely SpDF, aims to reduce the number of points while preserving as much as possible the topology of the point cloud using geometric primitives (i.e., curve, surface, and junction). Note that it is not limited to plane, line and point as the tensor voting framework allows to detect more generic geometric primitives. A major challenge in robotics applications is the non-uniformity of scans acquired with lidar sensors. In fact most of sampling algorithms are designed for uniform point clouds. This problem is addressed by proposing a new efficient strategy to make the density uniform for each of the three geometric primitives we consider. SpDF can be divided into two main steps: (1) making the density uniform for each geometric primitive; and (2) labeling and rejecting outliers according to the confidence in the geometric information. A visual overview of the algorithm is given in Figure 2.

### 5.1. Making the Density Uniform

**Figure 4:**
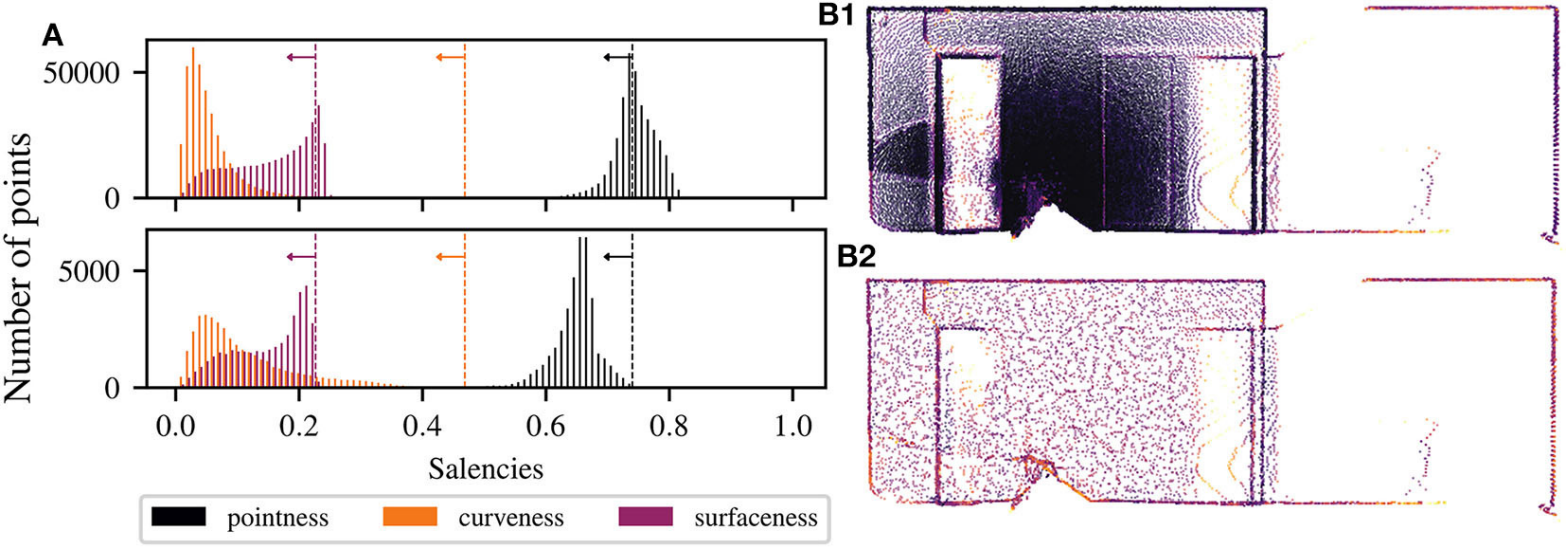
**(A)** Convergence of the saliencies below their expected values, represented by the vertical dashed lines, implying a uniform density on each geometric primitive. The used parameters are *k* = 50 and σ = ρ = 0.2. The top-histogram represents the initial saliencies distribution obtained from the tensor voting framework. The bottom-histogram shows the resulting distribution after making the density uniform. Illustration of the process of reducing and making uniform a structured point cloud from 370 k **(B1)** to 40 k points **(B2)**, i.e., a compression ratio of 89 %. The resulting point cloud have uniform density, and geometric primitives, such as corners, edges and surfaces, are preserved.

Using the new local density measure on each geometric primitive, the point cloud can be made uniform as follows. An iterative procedure allows to progressively decimate primitives where the saliencies are higher than the expected values reported in Table 2. The saliencies are recomputed using TV with tensors encoded as unit balls. The algorithm stops when the number of points is stable, which means that the saliencies distributions have converged below the expected values, as shown by Figure 4A. This figure shows the convergence of the saliencies below their expected values (represented by the vertical dashed lines), where the top-histogram represents the initial saliencies distribution and the bottom-histogram shows the resulting distribution after making the density uniform. Therefore, the densities are uniform around each primitive allowing us to detect them more clearly. Otherwise, most dense areas will be detected as junction because noise will predominate. An example of the result of making the density uniform (for *k* = 50 and σ = ρ = 0.2) is given by Figure 4B for a point cloud from 370 to 40 k points (i.e., a compression ratio of 89 %). The resulting point cloud have uniform density, and geometric primitives are preserved. All planes have then the same density, edges have been kept at the same density, and less points are wrongly identified as junction.

Finally, to control the density, the user only has to tune the parameter ρ, i.e., the radius of uniformity within the *D*-hyperball (where *D* is the dimension of the considered geometric primitive). Indeed, the expected vote strengths ξ_*D*_ is normalized, therefore σ (the spatial extent of the neighborhood) and *k* (the number of points to consider in the neighborhood) do not influence the density once set. In our experiment we set σ = 20 cm, *k* = 50, which is a good compromise between precision and time complexity, and ρ varies from 10 to 1.35 m. It means that once sampled, we expect a density of *k* points uniformly distributed within a ball of radius ρ for junctions, within a disk of radius ρ for surfaces, and on a segment of half-length ρ for curves.

### 5.2. Rejecting Outliers and Identifying Geometric Primitives

Given the saliencies computed with a last iteration of TV with the ball component disabled, each point is then labeled into *junction, curve*, or *surface* following the TV methodology, and the saliency associated (respectively point-ness, curve-ness,or surface-ness) encodes the confidence in this labeling. It provides a high level description in terms of geometry. Points with a confidence higher than *t* % of the maximum confidence of the considered geometric primitive are kept (we used in our experiments *t* = 10 %). This heuristic allows to reject outliers having a low confidence in their measure. At the end of the process, the point cloud is uniform, outliers have been rejected, and each point is labeled as surface, curve or point. SpDF is then able to reduce the point cloud while enhancing it with geometric information.

## 6. Experimental Setup

To validate our sampling method, we investigate its impact on a 3D registration process, and evaluate it along with several sampling methods. Indeed, registrations of point clouds is a mandatory step in most of robotics applications.

### 6.1. Registration Based on the Iterative Closest Point (ICP) Algorithm

Registration is the process of aligning the frames of two point clouds, the reference ***P*** and the reading ***Q***, by finding the rigid transformation ***T*** ∈ SE(3) between them by a minimization process. The transformation can be determined through the ICP algorithm introduced by Besl and McKay (1992) and Chen and Medioni (1992). The original algorithm only works well in ideal cases. To improve the robustness of the original formulation, several variants have been proposed. In order to leverage geometric information, such as normals, Rusinkiewicz and Levoy (2001) introduced an efficient variant of the ICP algorithm based on the *point-to-plane* formulation of the error minimization. Segal et al. (2009) proposed a generalization of the ICP algorithm, called Generalized-ICP, which takes into account the locally planar structure of both scans in a probabilistic model. Stoyanov et al. (2012) presented a novel approach to point cloud registration, based on minimizing the distance between Gaussian distributions. Point set are locally represented by their Normal Distribution Transform (NDT), which are a special case of Gaussian Mixture Model for representing the probability to find a surface point at a specific position in space. Pomerleau et al. (2015) highlight that surface reconstruction is expensive to compute, but at the same time highlight the fact that leveraging geometric information when using *point-to-plane* error leads to a faster convergence rate. Most of the current strategies rely on geometric information to minimize the error of the registration process, requiring then to process the point cloud to extract these information.

To evaluate the accuracy of the registration, we calculate separately the translation error part, ε_*t*_, and the rotational error part, ε_*r*_, the same way it is done by Pomerleau et al. (2013). Given the ground-truth transformation ***T***_*gt*_ and its corresponding transformation found by the registration solution ***T***, the remaining error Δ***T*** is defined as

(10) $$\begin{array}{l} \Delta T=\left[\begin{array}{cc} \Delta R & \Delta t\\ 0 & 1 \end{array}\right]=TT_{gt}^{-1}\text{,} \end{array}$$

and ε_*t*_ is then given as the Euclidean norm of the translation Δ***t***, and ε_*r*_ is defined as the Geodesic distance directly from Δ***R***, such as:

(11) $$\begin{array}{l} \varepsilon_{t}=∥\Delta t∥\text{ and }\varepsilon_{r}=\arccos\left(\frac{\text{trace }\left(\Delta R\right)-1}{2}\right)\text{.} \end{array}$$

As ICP needs a prior for fine registration (i.e., the initial transformation $\hat{T}$) to compute the transformation between two point clouds, we applied a uniform perturbation on the ground-truth transformation ***T***_*gt*_ using Lie algebra, such that $\hat{T}=\exp\left(\varsigma \right)T_{gt}$, with **ς** ∈ 𝔰𝔢(3) and exp(·) being the standard matrix exponential. For our experiments, a perturbation sampled from a uniform distribution of 50 cm was applied on the translation, and from a uniform distribution of 20° on the rotation. During the data filtering step, we applied the evaluated filter on both the reading and the reference. The data association is conducted by matching the two closest neighbors. We rejected the outliers in the matching process according to a trimmed distance. We limited the scope our experiments to a *point-to-plane* version of ICP, as it tends to perform better in those datasets as highlighted by Pomerleau et al. (2013). The minimization process stops when the number of iterations reached 150, or when the differential translation error is less than 1 cm and the differential rotation error is less than 0.001 rad.

### 6.2. Details on the Real-World Datasets

Working under the hypothesis of robotics applications, this paper presents an in-depth evaluation of sampling algorithms on (1) structured with 45 pairs of scans, (2) semi-structured with 32 pairs of scans, and (3) unstructured point clouds with 32 pairs of scans, using the datasets “Challenging data sets for point cloud registration algorithms” (Pomerleau et al., 2012)^2^. The structured environment is a map of an apartment with approximate dimension of 17 × 10 × 3 m, with an average 365 k points per scan. The semi-structured environment is a map of a park where there is grass, paved small roads, sparse trees, and containing a gazebo made of rock walls and a wood ceiling covered with vines trees. The dimension are 35 × 45 × 16 m, with an average of 170 k points per scan. Finally, the unstructured environment is a map of a wood, mainly constituted of vegetation (tree, bushes, etc.). The only structured element is a small paved road that crosses the wood. The dimension are 36 × 60 × 22 m, with an average of 178 k points per scan. The used sensor is an Hokuyo UTM-30LX, a time-of-flight sensor with a minimum range of 0.1 m and a maximal range of 30 m. As indicated by Pomerleau et al. (2012), the specifications of the sensor give a range accuracy varying from ± 1 cm for distances within [0.1, 10) m to ± 3 cm for distances within [10, 30] m.

### 6.3. Overview of the Evaluated Methods

To evaluate the impact of the number of points on the registration process, several methods from the state-of-the-art had been implemented in the open-source modular library for ICP named libpointmatcher, introduced by Pomerleau et al. (2013) and available online^3^. The eight evaluated filters, summarized in Table 3, are the following:

- Random sampling, chose as our baseline for its simplicity and because it is still one of the most used solution; point cloud is reduced by dropping points given a fixed probability.
- One neighbor-based method from the libpointmatcher, the Sampling Surface Normal filter, SSNormal; it recursively decomposes the point cloud into boxes until each box contains at most a given number of points, and select one point if normal estimation can be conducted within the box.
- Two variants of spatial segmentation, Voxel and Octree (centroid); Voxel sub-divides the space with a fixed spatial extent whereas Octree uses the number of points per cell as a criterion to stop the partition. Both take the centroid in each cell to sub-sample the point cloud.
- Two normal-based methods, CovS, the Covariance Sampling method from Gelfand et al. (2003) and NSS, the Normal Space Sampling method from Rusinkiewicz and Levoy (2001).
- Two density-based method, the MaxDensity method from the libpointmatcher and eventually, the proposed method in this paper, SpDF; MaxDensity aims to homogenize the density of a point cloud by rejecting a sub-sample of points in high-density regions. Points are only considered for rejection if they exceed a density threshold, otherwise they are preserved. It relies on a spherical approximation to compute density. SpDF also aims to homogenize the density but on each different geometric primitive. It leverages the tensor voting framework to identify these primitives and to derive density measures for each. It therefore reduces the number of points and enhances the point cloud with geometric information, such as the normals. Hence, SpDF can be seen as an improvement of the MaxDensity method.

**Table 3:** Ranges [*a*; *b*] of parameters influencing the number of points, where *a* gives the smallest number of points (here, 1,000 points) and *b* preserves all the points (with *n* being the total number of points), for each evaluated method with their description.

**Method**	**Parameter description**	**Range**
Random (baseline)	prob. to keep point	[0.004 ; 1.]
NSS	nb. of points to keep	[1000 ;*n*]
CovS	nb. of points to keep	[1000 ;*n*]
SSNormal	nb. of neighbors to merge	[253 ; 3.]
Octree	nb. max of points by cell	[1000 ; 1.]
Voxel	size max of the cell in m	[2.49 ; 0.001]
Max Density	nb. max of points by m^3^	[16.8 ; 506 k]
(SpDF) (ours)	radius of uniformity in m	[1.35 ; 0.1]

The evaluated methods are similar to the most used strategies available in point cloud processing software such as CloudCompare and PCL. For each method, we resumed in Table 3 the range [*a*; *b*] of parameters influencing the number of points, where *a* gives the smallest number of points, here, 1.000 points, and *b* preserves all the points, with *n* being the total number of points. We performed 2.500 registrations using the ICP algorithm (5.000 for our baseline Random) across the range for each method on each pair of scans, for each dataset, accumulating more than 2.45 million registrations in different types of environments.

## 7. Results and Discussion

### 7.1. Evaluation on Registration Accuracy

**Figure 5:**
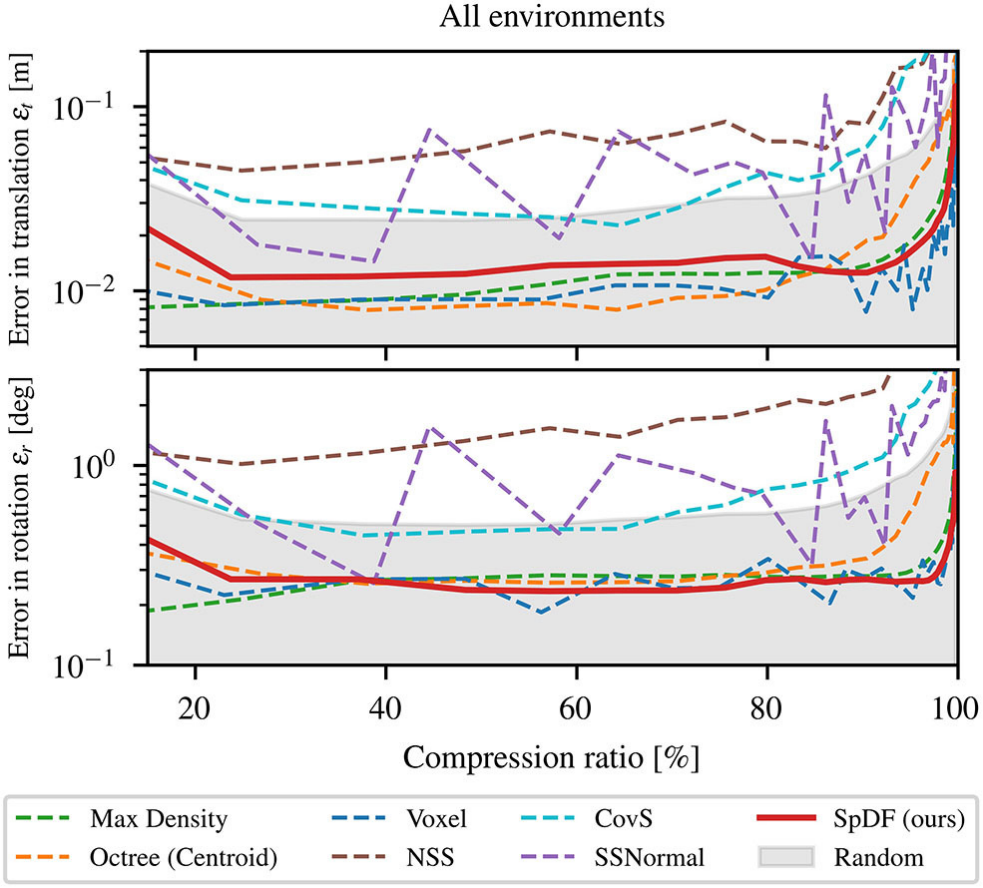
Influence of the number of points (i.e., compression ratio) on the registration process for all environments concatenated. The gray area represents the errors less than the baseline. SpDF (ours) is displayed in solid-line. The top-graph represents the error in translation ε_*t*_ in m; The bottom-graph represents the error in rotation ε_*r*_ in deg. Both translation and rotational errors show the same patterns. Only spatial methods (Voxel and Octree) and density-based methods (MaxDensity and SpDF (ours)) outperform the baseline, providing a translation error less than 2 cm, and a rotation error of less than 0.3°, which tend to the minimal reachable errors for the ICP algorithm.

First, we compare the different sampling methods on the registration accuracy. Figure 5 presents the translation and the rotational errors as functions of the compression ratio of the point clouds for all environments concatenated. At 0 %, all points have been kept and at 100 %, all points have been removed. The gray area represents the errors lower than our baseline (Random), the red solid-line corresponds to SpDF and the dashed-lines to the other methods.

Both translation (ε_*t*_) and rotational (ε_*r*_) errors show the same patterns. From 0 to 25 %, corresponding to more or less 100 k points, the errors decrease as the compression ratio increases. From 25 to 80 %, the errors are almost constant or grow at a really slow rate. Beyond 80 %, the errors start to increase exponentially. In the first situation, with more points, the dense areas predominate in the minimization process, leading to less accurate results. In the last situation, with only a few points, the minimization process cannot converge efficiently as not enough information is available to constrain the process. Both situations lead to less accurate results. The evaluated methods are significantly more accurate than the baseline except NSS, CovS, and SSNormal, which perform worse than Random, with a median error of 20 cm against 7 cm for the translation. This confirms their inability to manage uneven density and large-scale point cloud performing poorly for all types of environment. These algorithms need to be adapted for an application in the context of robotics, with large-scale, uneven environments.

Spatial methods perform well on all datasets, providing a median translation and rotation errors which tend to the minimal reachable errors for the ICP algorithm on these kind of maps. However, Octree starts diverging sooner than the others, around 90 %, corresponding to approximately 30 k points, when the number of points decreases. With Octree, we obtained a translation error of 1.9 cm and a rotational error of 0.4°. It performs well to a certain extent as it is able to preserve the spatial distribution for a large number of points, but suffers from the uneven density distribution for a small number of points. The Voxel method performs well as it preserves the spatial distribution whatever the density, with a translation error of 1.2 cm and a rotational error less than 0.27°. Using only the density, MaxDensity leads to more accurate alignments than the baseline showing a translation error of 1.6 cm and a rotational error less than 0.3°. Finally, SpDF with an error in translation of 1.8 cm and an error in rotation of 0.278°, shows the best results from all the evaluated methods along with MaxDensity and Voxel, which are equivalent in terms of errors. The proposed method inherits the qualities of the density-methods and manage efficiently large point clouds with uneven densities by maintaining a given density for each geometric primitive.

**Figure 6:**
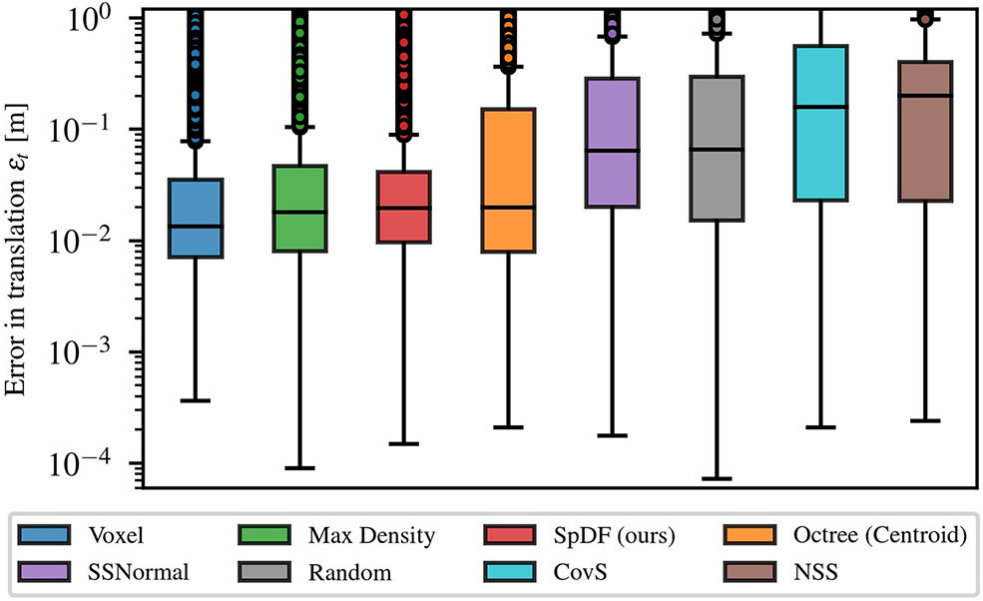
Statistic analysis of the translation error at compression ratio between 0 and 99.9 % for each method, graphically represented as box plots. Medians are given with their first $Q_{1}$ and third $Q_{3}$ quartiles. The spacing between the different parts of the box indicate the degree of dispersion (spread) and skewness in the errors. Boxes are given with whiskers with maximum 1.5 IQR, where $\text{IQR}=Q_{3}-Q_{1}$ is the inter-quartile range. We can clearly identify two clusters of solution: (1) Voxel, MaxDensity, and SpDF; and (2) SSNormal, Random, CovS, and NSS. The Octree method is in-between as the spread extends from the first to the second cluster.

Figure 6 gives a statistic analysis of the translation error at compression ratio between 0 and 99.9 % for each method, graphically represented as box plots. Medians are given with their first $Q_{1}$ and third $Q_{3}$ quartiles. The spacing between the different parts of the box indicate the degree of dispersion (spread) and skewness in the errors. Boxes are given with whiskers with maximum 1.5 IQR, where $\text{IQR}=Q_{3}-Q_{1}$ is the inter-quartile range. Points outside the boxes represent the outliers. We can clearly identify two clusters of solutions: (1) Voxel, MaxDensity, and SpDF; and (2) SSNormal, Random, CovS, and NSS. The Octree method is in-between as the spread extends from the first to the second cluster. The second cluster presents a median error between 6.5 and 20 cm with a large spread of more than 50 cm. The first cluster presents a median error between 1.2 and 1.8 cm but with an equivalent degree a dispersion of 2.8 cm. The three methods are therefore equivalent in terms of error. In the following, we will conduct the analysis only on the first cluster of solutions, i.e., on SpDF along with MaxDensity and Voxel.

### 7.2. Comparison for High Compression Ratio and for Each Type of Environment

**Figure 7:**
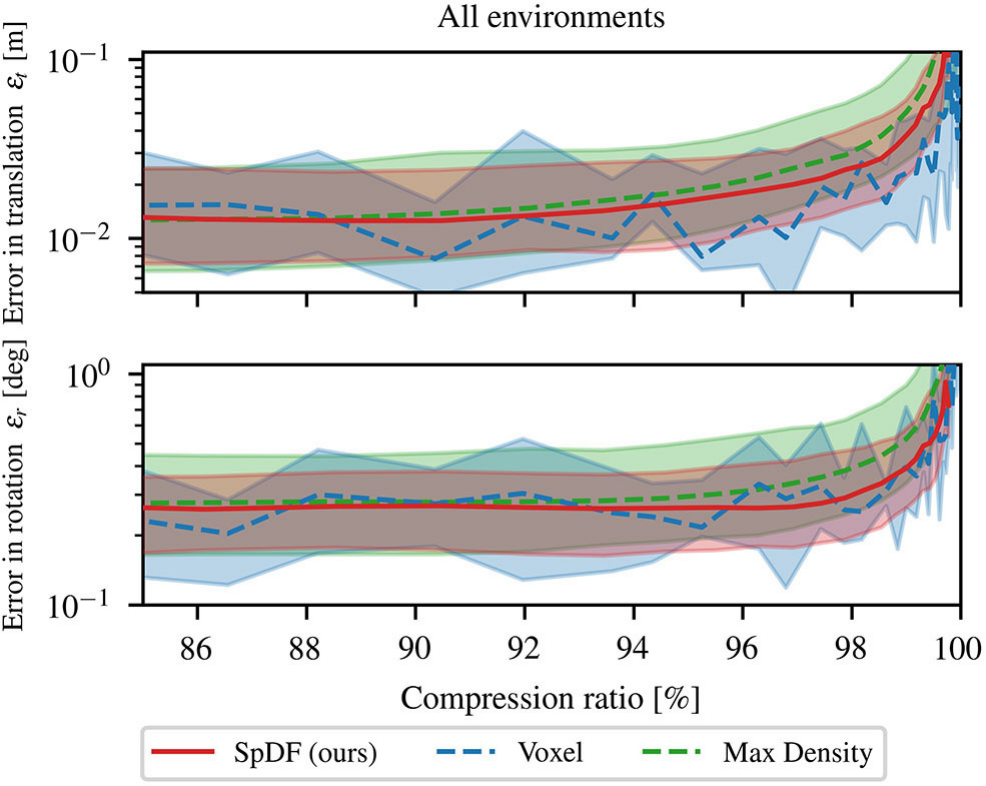
Comparison of Voxel, MaxDensity, and SpDF (ours) influence of the number of points on the registration process for high compression ratios. The top-graph represents the median error in translation ε_*t*_ in m. The bottom-graph represents the median error in rotation ε_*r*_ in deg. Both errors are displayed with their quartiles at 25 and 75 %. All method errors are of the same magnitude and can be considered equivalent. However, SpDF is slightly more stable as the compression rate grows compared to Voxel. We can expect a compression ratio of 97 % without hindering the registration process for all of these methods, i.e., ε_*t*_ < 2 cm and ε_*r*_ < 0.3°.

Secondly, we analyze the behavior for high compression ratio. The three best methods have been compared for compression ratio between 85 and 99.9 %. Errors in translation and rotation are illustrated by Figure 7. Both errors are displayed with their quartiles at 25 and 75 %. All method errors are of the same magnitude, even if MaxDensity performs slightly worse as the compression ratio grows. Rotational error is constant and less than 0.3° until 97 % and start growing exponentially afterward. The same behavior is observed for the translation errors which are less than 2 cm until a compression of 92 %. We believe that the error tends to the minimal reachable error that can be obtained with the ICP algorithm, making it hard to state that one method outperforms the others. However, SpDF and MaxDensity are slightly more stable as the compression rate grows compared to Voxel. One explanation is that spatial sampling depends on the origin of the sampling and on the frequency: the larger the spatial extent of the voxel, the greater the shift in the point cloud. Sampled point in the reading and reference point cloud might not represent the same part of the point cloud, leading to less stable results while matching. Finally, density-based methods as ours are able the perform well in context of 3D registration because they are able to deal with noisy and non-uniform point clouds.

**Figure 8:**
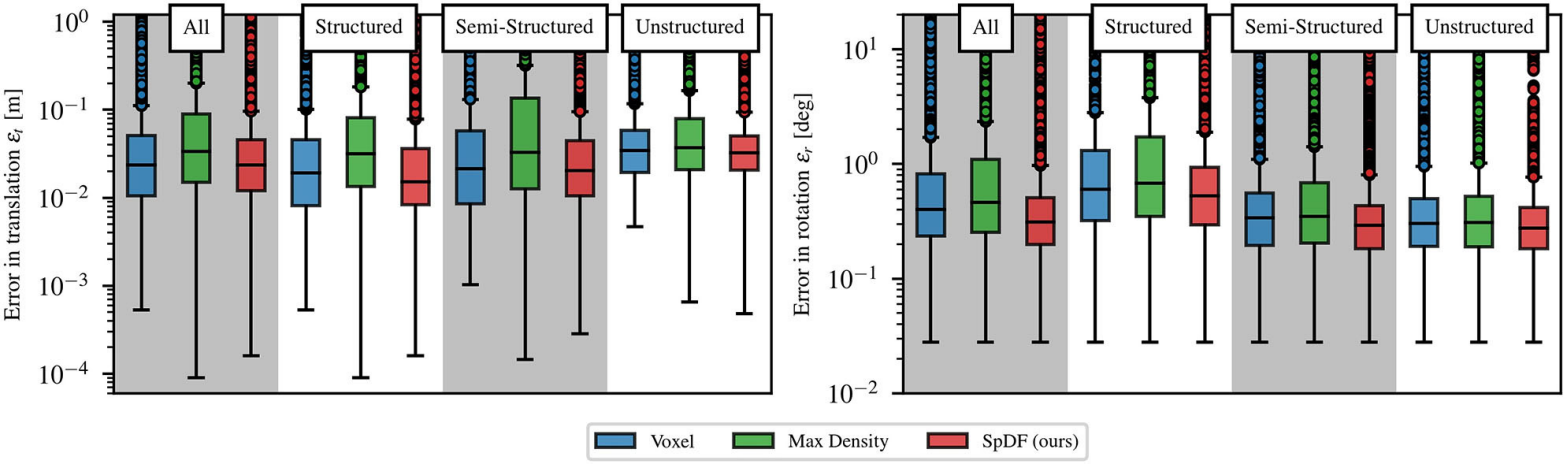
Statistic analysis of the translation and rotation errors at high compression ratio between 85 and 99.9 % for each environment (structured, semi-structured, unstructured and all environments concatenated), for the top three methods, graphically represented as box plots. Medians are given with their first $Q_{1}$ and third $Q_{3}$ quartiles. The spacing between the different parts of the box indicate the degree of dispersion (spread) and skewness in the errors. Boxes are given with whiskers with maximum 1.5 IQR, where $\text{IQR}=Q_{3}-Q_{1}$ is the inter-quartile range. All environments present similar errors, except in rotation for the structured environment which presents a slightly higher error. The spreads and medians are of the same order for the three methods.

These three methods are equivalent in terms of translation and rotation errors. Statistical analysis reported in Figure 8 confirms this hypothesis. On this range of compression ratio, we can expect a translation errors of: 2.37 cm with a lower quartile $Q_{1}=$ 1.05 cm, and upper quartile $Q_{3}=$ 5.11 cm, i.e., an IQR of 4.06 cm for Voxel; for SpDF, an error of 2.40 cm with a lower quartile $Q_{1}=$ 1.21 cm, and upper quartile $Q_{3}=$ 4.59 cm, i.e., an IQR of 3.37 cm; and of 3.59 cm with a lower quartile $Q_{1}=$ 1.63 cm, and upper quartile $Q_{3}=$ 9.62 cm, i.e., an IQR of 7.99 cm for MaxDensity. MaxDensity provides a higher error and spread than the two others methods. Voxel and SpDF are equivalent in terms of median errors, but SpDF is slightly more stable as the spread is lower. On this range of compression ratio, we can expect a rotation errors of: 0.40° with a lower quartile $Q_{1}=$ 0.23°, and upper quartile $Q_{3}=$ 0.82°, i.e., an IQR of 0.59° for Voxel; for SpDF, an error of 0.31° with a lower quartile $Q_{1}=$ 0.19°, and upper quartile $Q_{3}=$ 0.50°, i.e., an IQR of 0.31°; and of 0.48° with a lower quartile $Q_{1}=$ 0.26°, and upper quartile $Q_{3}=$ 1.15°, i.e., an IQR of 0.89° for MaxDensity. Compared to MaxDensity and Voxel, SpDF gives the lowest median error with the smallest spread, but the difference is not significant enough and all three methods can be considered equivalent.

Thirdly, we analyze the impact of the type of environment on the registration error for high compression ratio between 85 and 99.9 %. Figure 8 gives a statistic analysis of the translation and rotation error for the top three methods, graphically represented as box plots. Medians are given with their first $Q_{1}$ and third $Q_{3}$ quartiles. Boxes are given with whiskers with maximum 1.5 IQR. Even if each environment shows its own kind of errors variations, the methods behave similarly independently of the types of environment. For the rotation errors, the semi-structured and unstructured present similar errors of 0.3°, with similar dispersion of 0.3°. On the structured environment, the evaluated methods perform slightly worse, with an error of 0.6° with a larger spread of 0.8°. One explanation is that the latter environment usually presents a non-homogeneous distribution of the surfaces orientations (some orientations are preferred, e.g., the walls orientations) leading to less precise rotation estimation. One another explanation is that the structured environment is at a smaller scale than the others, which leads to a noisier estimation of the rotational component. Translation errors are of the same order for all the environments. We can expect an error of less than 3 cm with a 5 cm dispersion. The spread is smaller in the case of the unstructured environment than the other two. In particular, the spread of the MaxDensity is slightly higher than the others. Unsurprisingly, translation median errors are greater for the unstructured than the structured environment, as the *point-to-plane* cost function performs better in latter environments.

### 7.3. Computation Time Analysis

**Figure 9:**
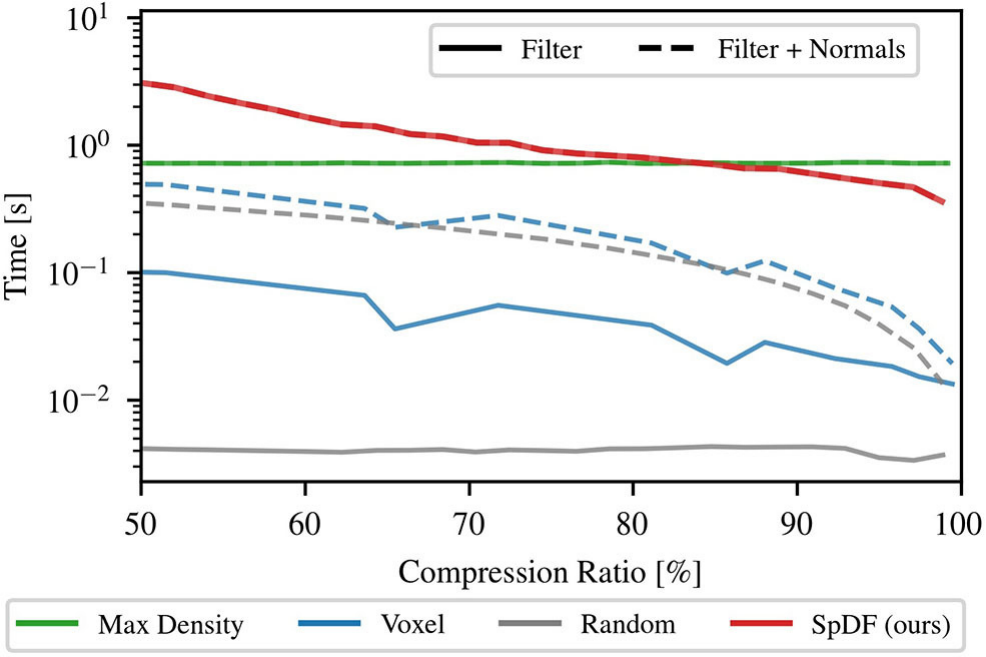
Computation time analysis as function the compression ratio for Random, Voxel, MaxDensity, and SpDF (ours) sampling methods. The computation time of applying the filter (solid-line) has been measured along with the accumulated time required for normals estimations (dashed-line). For MaxDensity and Random, the computation time of applying the filter is constant with respect to the compression ratio. For Voxel and SpDF, it decreases while the compression ratio increases, as the number of cells decreases and the number of iterations for the voting process also decreases.

Computation time analysis has been conducted on an Intel^®^ Core™ i7-7820HQ CPU @ 2.90GHz, with 8 cores, on Ubuntu 16.04.3. Parallelization has been enabled when possible using then 8 threads. Figure 9 provides a comparison of the computation time as function of the compression ratio, from 50 to 99.9 %, for the following methods: Random, MaxDensity, Voxel, and SpDF. Voxel construction and the voting process have been parallelized. For each method, 2.500 samples have been used to sub-sample one scan per type of environment. The three types of environments are: structured, with 198 k points; semi-structured, with 134 k points; and unstructured, with 166 k points. The results are presented for all environments concatenated.

First, only the computation time of applying the filter has been measured, therefore not including the time required by the ICP process. As shown in Figure 9, the computation time of MaxDensity and Random is constant with respect to the compression ratio. Random iterates only once on all the point cloud to decimate randomly the points, and MaxDensity computes the densities using the full point cloud, then randomly removes points with high density. For Voxel and SpDF, the computation time decreases while the compression ratio increases, as the number of cells decreases and the number of iterations for the voting process also decreases. Due to its iterative nature, SpDF is more costly and slower: for low compression ratios, one order of magnitude against MaxDensity, two order against Voxel, and three order against Random; for high compression ratios, MaxDensity is of the same order, one order against Voxel and two order against Random. For instance, at a compression ratio of 95 %, for the semi-structured environment, we have the following computation times: *t*_Random_ = 4.5 ms, *t*_Voxel_ = 20 ms, *t*_MaxDensity_ = 750 ms, and *t*_SpDF_ = 500 ms. The evaluated strategies do only reduce the input point cloud, while SpDF is designed to reduce and enhance the point cloud with geometric information at the same time. Therefore, it is clear that the method has a higher computational time, but what we lose in computation time is gained in geometric information. As the computation time tends to the limit of real-time capabilities, efforts might be done to optimize the code and could be ported to GPU. Especially, porting the tensor voting and tensor decomposition parts on GPU, as done in Liu et al. (2012), provides significant improvement in computation time.

Secondly, considering the computational time of the whole pipeline of the ICP algorithm, we need to take into account the pre-processing time. In the case of the *point-to-plane* version it is required to compute the normals. Figure 9 presents for each method the accumulated time needed for applying the filter and computing the normals. Normals estimation is conducted on the sampled point cloud. For MaxDensity, normals estimation is performed at the same time as the densities calculations, therefore there is no additional time. SpDF intrinsically computes the normals when applied, hence requiring no additional time either. For the other methods, the computation time is significantly increased for low compression ratio. At a compression ratio of 50 %, Random required a total computation time of 350 ms against 4.5 ms without normals estimation. The computation time of Voxel increases from 100 to 500 ms. The time required is now of the same order of magnitude as MaxDensity. The gap between Voxel and SpDF is now only of a factor 6. For high compression ratio, the difference is less significant. At a high compression ratio of 90 %, Random required a total computation time of 100 ms. The computation time of Voxel increases up to 200 ms, which is however still one order of magnitude quicker than SpDF. Note that when not enough points are available, the normals estimation is not robust and could hinder the registration process. SpDF leverages the tensor voting framework to provide a robust estimate of the normals.

### 7.4. Preserving Details and Making the Density Uniform on Real-World Large-Scale Data

Finally, we evaluate qualitatively SpDF on real-world large-scale datasets. Figure 4 illustrates that SpDF is able to make the density uniform while preserving each geometric primitive, from an original point cloud of 370 k points where most points are concentrated in a small area, to a uniform point cloud of 40 k points, i.e., a compression ratio of 89 %. Edges and corners have been preserved, while dense surfaces have been made uniform.

We evaluated the proposed method along with one of the top three methods, Voxel sampling with a spatial extent of 0.8 m on a real-world large-scale outdoor environment. Figure 1 shows the qualitative result of a sampled point cloud from 4.65 million points to 100 k points, i.e., a compression ratio of 97.85 %. The map dimensions are approximately of 340 × 100 × 6 m. Figure 1B2 gives the full and top views of the map sampled by Voxel. The spatial sampling stops the voxel decomposition when the cell size is less than 0.8 m or when the cell contains less than 45 points. The decomposition is aligned with the *xyz*-basis. In this case, Voxel suffers from the discrepancy between the *x*, *y*, and *z* scales, as it creates the bounding box with a radius of the size of the bigger dimension. Furthermore, as the ground is not aligned with the *xy*-plane, it keeps points which are at different altitudes within the dense path. Finally, when geometric details are not dense enough such as the pillars of the top-left of the map, Voxel does not keep them. Contrarily, SpDF identifies the geometric primitives, and is able to sub-sample efficiently each primitive locally: the ground is uniformly sampled, and the pillars of the top-left of the map are preserved even if the density is low. From Figure 1B1, the full and top views of the map sampled by SpDF show that most of the details have been preserved and the density is uniform.

**Figure 10:**
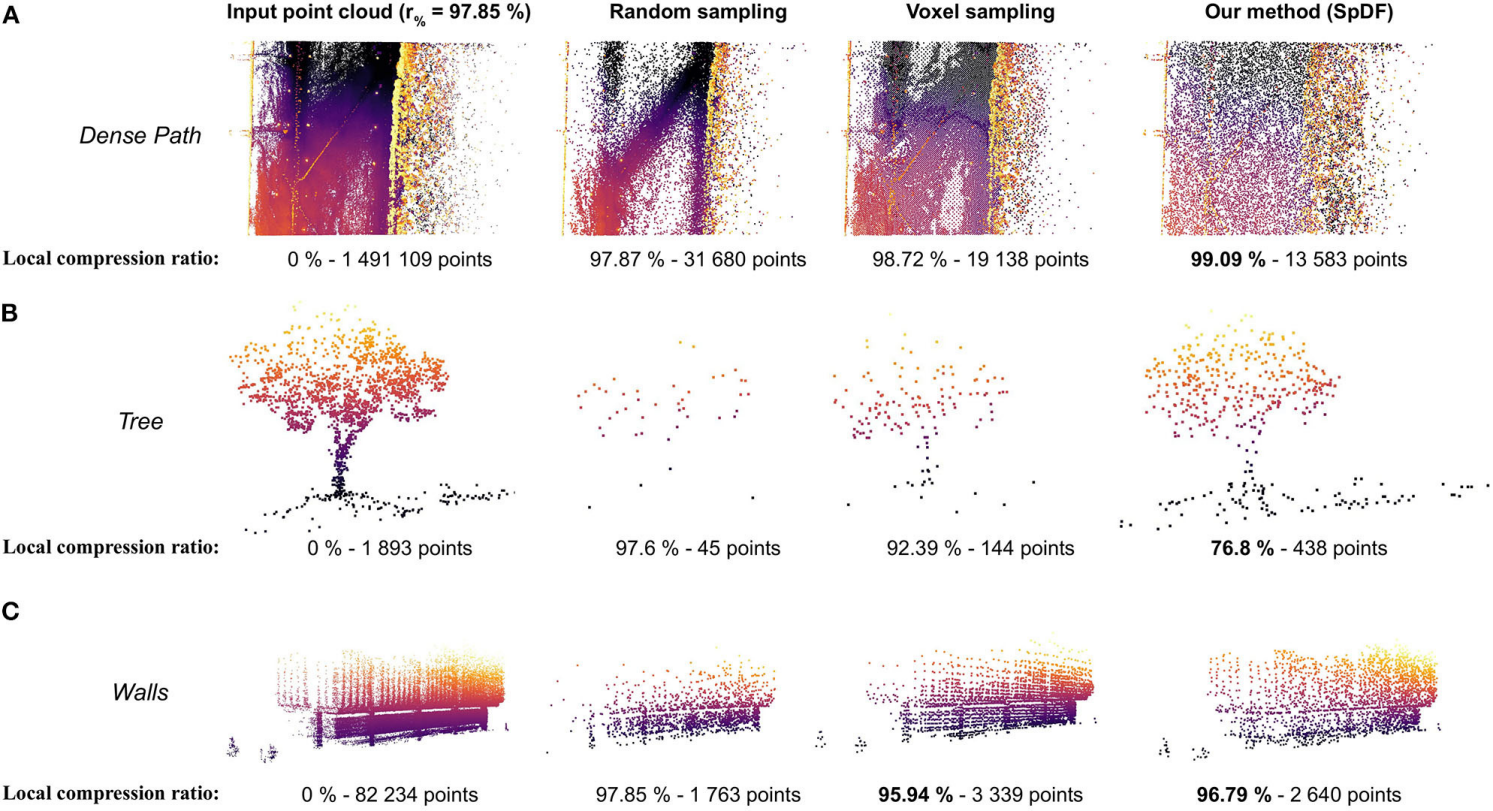
Sub-views comparison from the *Grand Axe* map, containing more than 4.65 million points with varying densities. The whole point cloud has been sampled to 100 k points (i.e., a compression ratio of 97.85 %) by Random sampling, by Voxel sampling and by our method, SpDF. Local compression ratio has been reported for each sub-view. **(A)** Sub-view of a dense path containing a very high density of points. Random kept a lot of points and still presents varying densities. Voxel regularly sampled the path, but kept slightly more points as the ground is not aligned with the *xy*-plane. SpDF aggressively sampled the path, adapting then the local compression ratio up to 99 %, and have made the density uniform. **(B)** Sub-view of one tree. With Random, most of the points have been removed due to low local density and the tree is no longer distinguishable, while with SpDF, we efficiently sample the tree, reducing then the local compression ratio to 76.8 %, and keep the geometric information. Voxel kept slightly less points than SpDF, preserving less details. **(C)** Sub-view of structured walls. Random kept more points in the dense part of the wall, losing then the geometric information of the upper part. Voxel kept the more point, regularly sampled the walls but loose the original geometric details. It removed the structured elements with low density on the top-left, while our method, SpDF, kept points uniformly on the structure, reducing then the local compression ratio to 96.79 %.

Figure 10 illustrates the effects of the proposed method on large-scale point cloud, presenting a comparison of sub-views from the *Grand Axe* map. Sub views have been extracted from the whole sampled point cloud by Random sampling, by Voxel sampling and by SpDF. Local compression ratio has been reported for each sub-view. Figure 10A presents a sub-view of a dense path containing a very high density of points. Random kept a lot of points and still presents varying densities. Voxel regularly sampled the path, but still kept slightly more points as the ground is not aligned with the *xy*-plane. SpDF aggressively sampled the path, adapting then the local compression ratio up to 99 %, and have made the density uniform. Figure 10B is a sub-view of one tree representing less than 0.04 % of the total point cloud. With Random, most of the points have been removed due to the low local density and the tree is no longer distinguishable. Voxel kept more points than Random, allowing to identify the tree within the point cloud, reducing the compression ratio to 92.39 %. However, less details have been preserved, and most of the ground have been removed. With SpDF, we efficiently sample the tree, reducing then the local compression ratio to 76.8 %, and keep most of the details. Finally, Figure 10C gives a sub-view of structured walls. Random kept more points in the dense part of the wall, then losing the geometric information of the upper part. It also removed the two small bushes next to the building, which are kept by the other methods. Even if Voxel sampled regularly the walls and kept more points, it does not differentiate the geometric primitives. For instance, structured elements have not been kept but replaced by regular samples, and the ones with a low density (on the left) have been removed. SpDF kept points uniformly on the structure, reducing then the local compression ratio to 96.79 %.

### 7.5. Discussion on the Choice of the Sampling Method

Our results show that sampling strategies designed for computer graphics applications cannot be extended to real-world large dataset. CovS and NSS cannot handle large scale map with noise, while SSNormal and Random suffer from their inability to deal with varying densities.

Considering real-world large scale environments with spurious and noisy measurements, Voxel, MaxDensity, and SpDF can be used to sample the point cloud. In applications where computation time is critical, Random can still be considered as a strong solution. However, if the precision is critical, it is better to use Voxel, MaxDensity, or SpDF as they provide errors within the range accuracy of the used sensor. Here, in the context of large scale environments, it represents less than 2 cm in translation and less than 0.3 deg in rotation up to a compression ratio of 97 %. The Voxel method provides an efficient representation of the map by sampling regularly the point cloud space, with a computational time of less than 100 ms. However, one drawback of the spatial methods is the lack of fine grained control over the number of output points and over the level of details. In applications where geometric information is required, such as surface inspection scenario, Voxel does not guarantee to preserve the geometric details as it averages the points within each voxel. Density-based methods, such as MaxDensity are able to preserve further geometric information as they work locally. However, MaxDensity does not differentiate geometric primitive during the density calculation and relies on spherical approximation. Our qualitative experiments show that SpDF by sampling locally on each geometric primitive further preserves the details and the topology of the point cloud. The major drawback of the proposed method is its high computational time at the limit of real-time capabilities, but does not required a pre-processing step. It provides a higher level description in terms of geometric primitives that could be used by another pipeline for scene interpretation, or directly in the minimization process as normals are provided in the *point-to-plane* version.

The general registration strategy selected for an application may require to balance advantages and drawbacks of the chosen solutions. Readers are invited to refer to Pomerleau et al. (2015) as they provide a clear overview of geometric registration in case of robotics applications, as well as a method to solve it in specific instances. Typical use-cases for ICP do not need to apply the filter at a high rate on all input scans. Most of the strategies rely on scan-to-map registration, where the maintained map is sup-sampled by an efficient sampling strategy according to the desired application. For instance, in case of surface inspection, we want to preserve geometric information, we can then use the proposed method in this paper. At 10 Hz, input scans should use Random to maintain high rates. Reducing the map size increases all registration done to it and decreases data transmission bandwidth when it comes to sharing the map.

## 8. Conclusion

This paper presents a novel sampling algorithm, named Spectral Decomposition Filter (SpDF), aiming at reducing the number of points while preserving the geometric information along the topology of large-scale point cloud with non-uniform density, large sensor noise, and spurious measurements. This method builds on spectral decomposition applied to point clouds in order to obtain a density better suited for robotics applications where geometric information are essentials. First, we identify the geometric primitives along with their saliencies using the tensor voting framework from the input point cloud. Then, we derive density measures from saliencies: if the density for each geometric primitive is less than the desired density, we stop; else we sub-sample each geometric primitive, and re-iterate. As output, we have a uniform sampled point cloud enhanced with geometric information.

We verified the feasibility of our method through quantitative and qualitative results. We presented a large-scale evaluation of SpDF along with other seven point cloud sampling strategies from the state-of-the-art, in the context of the 3D registration problem using the ICP algorithm. Our results show that only spatial sampling strategies and density-based methods are able to manage such large 3D environments without hindering the registration process. In particular, SpDF performs successfully on large-scale maps acquired with lidar sensor, where the density is non-uniform. We manage non-uniform densities by leveraging the new derived measures of density from saliencies for each geometric primitive which allows us to preserve the topology of the point cloud. Thus, by making the density uniform and leveraging the geometric information, the proposed method efficiently sub-samples large scale point cloud while simultaneously enhancing it. Even for high compression ratio (i.e., > 90 %), the process of registration is not hindered which enable a large spectrum of robotics applications. Indeed, for such applications, this sampling is usually critical to reduce bandwidth or computation complexity without losing accuracy during the registration process, whilst geometric information provides higher level of description.

Computational time is however still high and at the limit of real-time capabilities. Future works will include efforts to optimize the code and the tensor voting part will be ported on GPU which should significantly improve the computation time (Liu et al., 2012). Furthermore, as we provide a second order symmetric tensor representation for each point (i.e., a Gaussian representation), future works will aim to leverage this information directly in the minimization process of the ICP, inspired by the *Point-to-Gaussian* cost function derived by Babin et al. (2019).

## Data Availability Statement

The raw data supporting the conclusions of this article will be made available by the authors, without undue reservation.

## Author Contributions

ML: conceptualization, methodology, software development, validation, formal analysis, visualization, and writing. JL: methodology, formal analysis, visualization, and writing. FP: supervision, review, and project administration. All authors contributed to the article and approved the submitted version.

## Conflict of Interest

The authors declare that the research was conducted in the absence of any commercial or financial relationships that could be construed as a potential conflict of interest.

## Footnotes

^1^The original formulation can be extended to *n*-D data (Tang et al., 2001).

^2^Details on dataset can be found online at https://projects.asl.ethz.ch/datasets/doku.php?id=laserregistration:laserregistration (last accessed July 27, 2020).

^3^The library is available at https://github.com/ethz-asl/libpointmatcher. The filters documentation is available at https://libpointmatcher.readthedocs.io/en/latest/DataFilters/ (last accessed August 06, 2020).

## References

[B1] Al-Durgham M. M. (2014). The Registration and Segmentation of Heterogeneous Laser Scanning Data (Ph.D. thesis). University of Toronto, Toronto, ON, Canada.

[B2] Alexa M. Behr J. Cohen-Or D. Fleishman S. Levin D. Silva C. (2001). Point set surfaces, in Proceedings Visualization, 2001. VIS '01 (San Diego, CA), 21–28.

[B3] Al-Rawabdeh A. He F. Habib A. (2020). Automated feature-based down-sampling approaches for fine registration of irregular point clouds. Remote Sens. 12:1224 10.3390/rs12071224

[B4] Babin P. Dandurand P. Kubelka V. Giguère P. Pomerleau F. (2019). Large-scale 3D mapping of subarctic forests, in Proceedings of the Conference on Field and Service Robotics (FSR). Springer Tracts in Advanced Robotics (Tokyo).

[B5] Besl P. J. McKay N. D. (1992). A method for registration of 3D shapes. IEEE Trans. Pattern Anal. Mach. Intell. 14, 239–256. 10.1109/34.12179116325826

[B6] Chen Y. Medioni G. (1992). Object modeling by registration of multiple range images. Image Vision Comput. 10, 145–155. 10.1016/0262-8856(92)90066-C

[B7] Cignoni P. Montani C. Scopigno R. (1998). A comparison of mesh simplification algorithms. Comput. Graph. 22, 37–54. 10.1016/S0097-8493(97)00082-4

[B8] Elseberg J. Borrmann D. Nüchter A. (2013). One billion points in the cloud – An octree for efficient processing of 3D laser scans. ISPRS J. Photogrammet. Remote Sens. 76, 76–88. 10.1016/j.isprsjprs.2012.10.004

[B9] Ervan O. Temeltas H. (2019). Downsampling of a 3D LiDAR point cloud by a tensor voting based method, in ELECO 2019 - 11th International Conference on Electrical and Electronics Engineering (Bursa), 880–884.

[B10] Fossel J. Tuyls K. Schnieders B. Claes D. Hennes D. (2017). NOctoSLAM: Fast octree surface normal mapping and registration, in IEEE International Conference on Intelligent Robots and Systems (IROS) (Vancouver, BC: IEEE), 6764–6769.

[B11] Gelfand N. Ikemoto L. Rusinkiewicz S. Levoy M. (2003). Geometrically stable sampling for the ICP algorithm, in Fourth International Conference on 3-D Digital Imaging and Modeling (Banff, AB), 260–267.

[B12] Guy G. Medioni G. (1997). Inference of surfaces, 3D curves, and junctions from sparse, noisy, 3D Data. IEEE Trans. Pattern Anal. Mach. Intell. 19, 1265–1277. 10.1109/34.632985

[B13] Han X. F. Jin J. S. Wang M. J. Jiang W. Gao L. Xiao L. (2017). A review of algorithms for filtering the 3D point cloud. Signal Process. Image Commun. 57, 103–112. 10.1016/j.image.2017.05.009

[B14] Hoppe H. (1996). Progressive meshes, in Computational Geometry (New York, NY: Association for Computing Machinery), 99–108. Available online at: https://dl.acm.org/doi/proceedings/10.1145/237170

[B15] Hornung A. Wurm K. M. Bennewitz M. Stachniss C. Burgard W. (2013). Octomap: an efficient probabilistic 3D mapping framework based on octrees. Auton. Robots 34, 189–206. 10.1007/s10514-012-9321-0

[B16] Kalogerakis E. Nowrouzezahrai D. Simari P. Singh K. (2009). Extracting lines of curvature from noisy point clouds. CAD Comput. Aided Design 41, 282–292. 10.1016/j.cad.2008.12.004

[B17] Kwok T.-H. Tang K. (2015). Improvements to the iterative closest point algorithm for shape registration in manufacturing. J. Manufact. Sci. Eng. 138:011014 10.1115/1.4031335

[B18] Kwok T. H. (2018). DNSS: Dual-Normal-Space Sampling for 3-D ICP Registration. IEEE Trans. Autom. Sci. Eng. 16, 241–252. 10.1109/TASE.2018.2802725

[B19] Labussière M. Laconte J. Pomerleau F. (2018). Geometry preserving sampling method based on spectral decomposition for 3D registration. arXiv:1810.01666v2. 10.3389/frobt.2020.572054 PMC7806074 33501332

[B20] Leal N. Leal E. German S. T. (2017). A linear programming approach for 3D point cloud simplification. IAENG Int. J. Comput. Sci. 44, 60–67. Available online at: http://www.iaeng.org/IJCS/index.html

[B21] Li Y. Zhu Q. (2008). A new mesh simplification algorithm based on quadric error metrics, in Proceedings - 2008 International Conference on Advanced Computer Theory and Engineering, ICACTE 2008 (Phuket), 528–532.

[B22] Liu M. Pomerleau F. Colas F. Siegwart R. (2012). Normal estimation for pointcloud using GPU based sparse tensor voting, in IEEE International Conference on Robotics and Biomimetics (ROBIO), 91–96.

[B23] Medioni G. Tang C. K. Lee M. S. (2000). Tensor voting: theory and applications, in Reconnaissance des formes et Intelligence Artificielle (RFIA) (Paris).

[B24] Mellado N. Aiger D. Mitra N. J. (2014). Super 4PCS fast global pointcloud registration via smart indexing. Eurogr. Sympos. Geomet. Process. 33, 205–215. 10.1111/cgf.12446

[B25] Oztireli C. Alexa M. Berlin T. Gross M. Zürich E. (2010). Spectral Sampling of Manifolds: Extended Version. Technical Report, ETH Zurich, Department of Computer Science.

[B26] Pauly M. Gross M. (2001). Spectral processing of point-sampled geometry, in Proceedings of the 28th Annual Conference on Computer Graphics and Interactive Techniques (New York, NY), 379–386.

[B27] Pauly M. Gross M. Kobbelt L. (2002). Efficient simplification of point-sampled surfaces, in IEEE Visualization, 2002. VIS 2002 (Boston, MA: IEEE), 163–170.

[B28] Pomerleau F. Colas F. Siegwart R. (2015). A review of point cloud registration algorithms for mobile robotics. Found. Trends Robot. 4, 1–104. 10.1561/9781680830255

[B29] Pomerleau F. Colas F. Siegwart R. Magnenat S. (2013). Comparing ICP variants on real-world data sets. Auton. Robots 34, 133–148. 10.1007/s10514-013-9327-2

[B30] Pomerleau F. Krusi P. Colas F. Furgale P. Siegwart R. (2014). Long-term 3D map maintenance in dynamic environments, in IEEE International Conference on Robotics and Automation (Hong Kong), 3712–3719.

[B31] Pomerleau F. Liu M. Colas F. Siegwart R. (2012). Challenging data sets for point cloud registration algorithms. Int. J. Robot. Res. 31, 1705–1711. 10.1177/0278364912458814

[B32] Rodolà E. Albarelli A. Cremers D. Torsello A. (2015). A simple and effective relevance-based point sampling for 3D shapes. Pattern Recogn. Lett. 59, 41–47. 10.1016/j.patrec.2015.03.009

[B33] Rusinkiewicz S. Levoy M. (2001). Efficient variants of the ICP algorithm, in Proceedings Third International Conference on 3-D Digital Imaging and Modeling (Quebec City, QC), 145–152.

[B34] Rusu R. B. Blodow N. Beetz M. (2009). Fast Point Feature Histograms (FPFH) for 3D registration, in 2009 IEEE International Conference on Robotics and Automation (Kobe: IEEE), 3212–3217.

[B35] Schnabel R. Klein R. (2006). Octree-based point-cloud compression, in Eurographics Symposium on Point-Based Graphics (Boston, MA), 111–120.

[B36] Segal A. V. Haehnel D. Thrun S. (2009). Generalized-ICP. Proc. Robot. Sci. Syst. 2:4 10.15607/RSS.2009.V.021

[B37] Stoyanov T. Magnusson M. Andreasson H. Lilienthal A. J. (2012). Fast and accurate scan registration through minimization of the distance between compact 3D NDT representations. Int. J. Robot. Res. 31, 1377–1393. 10.1177/0278364912460895

[B38] Stumm E. Breitenmoser A. Pomerleau F. Pradalier C. Siegwart R. (2012). Tensor-voting-based navigation for robotic inspection of 3D surfaces using lidar point clouds. Int. J. Robot. Res. 31, 1465–1488. 10.1177/0278364912461537

[B39] Tang C. K. Medioni G. Lee M. S. (2001). N-dimensional tensor voting and application to epipolar geometry estimation. IEEE Trans. Pattern Anal. Mach. Intell. 23, 829–844. 10.1109/34.946987 15742892

[B40] Wu T.-P. Yeung S.-K. Jia J. Tang C.-K. Medioni G. (2012). A closed-form solution to tensor voting: theory and applications. IEEE Trans. Pattern Anal. Mach. Intell. 34, 1482–1495. 10.1109/TPAMI.2011.250 22184257

[B41] Zhang K. Qiao S. Wang X. Yang Y. Zhang Y. (2019). Feature-preserved point cloud simplification based on natural quadric shape models. Appl. Sci. 9:2130 10.3390/app9102130

